# Supplementary figures and images for: Detecting fabrication in large-scale molecular omics data
Source: PLoS One. 2021 Nov 30;16(11):e0260395. doi: 10.1371/journal.pone.0260395 (PMC8631639; doi:10.1371/journal.pone.0260395)

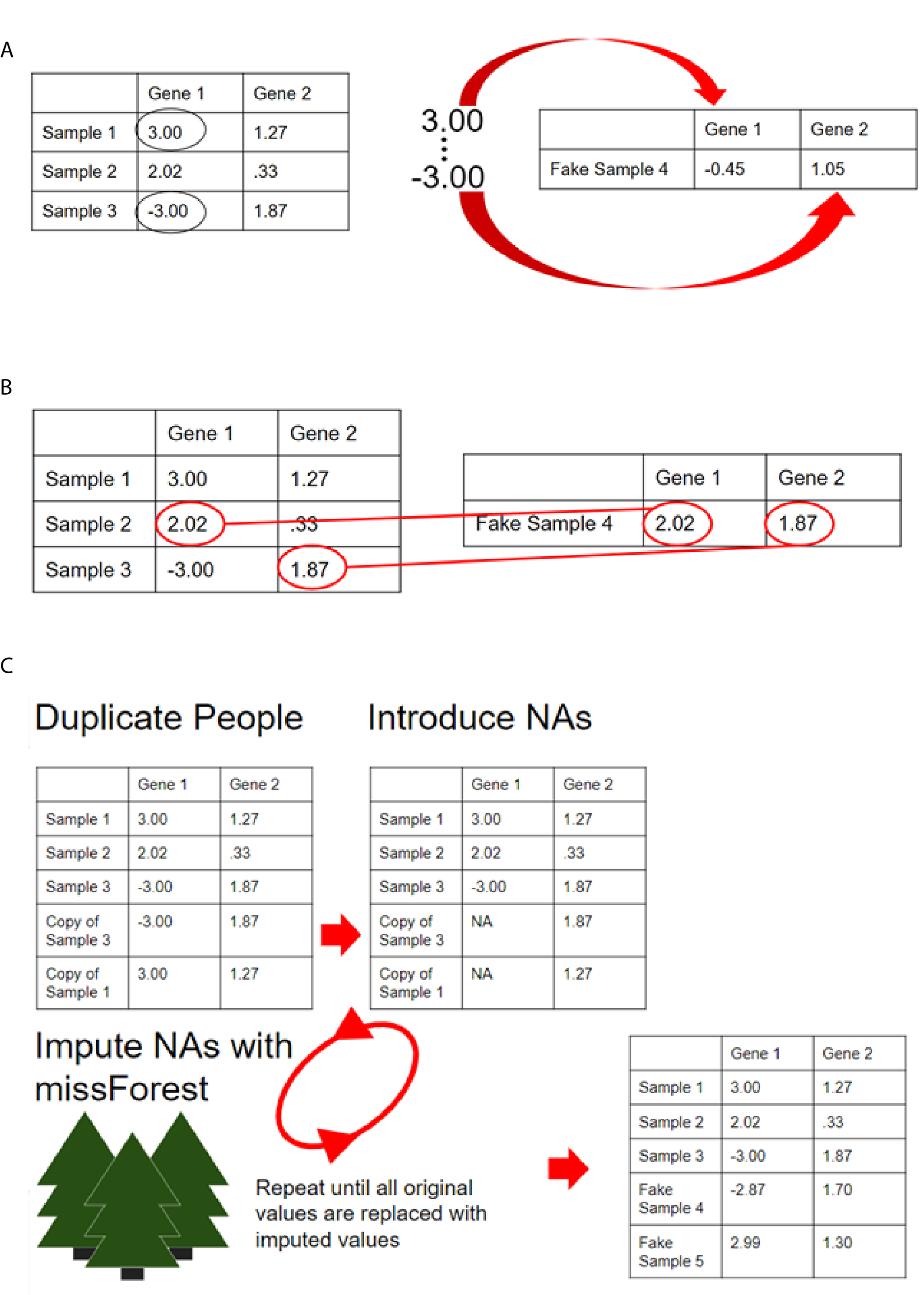

Supplement: S1 Fig — (A) The random method of data fabrication identifies the range of observation for a specific locus and then randomly chooses a number in that range. (B) The resampling method chooses values present in the original data. (C) The imputation method iteratively nullifies and then imputes data points from a real sample. (TIF) [file pone.0260395.s001.tif]

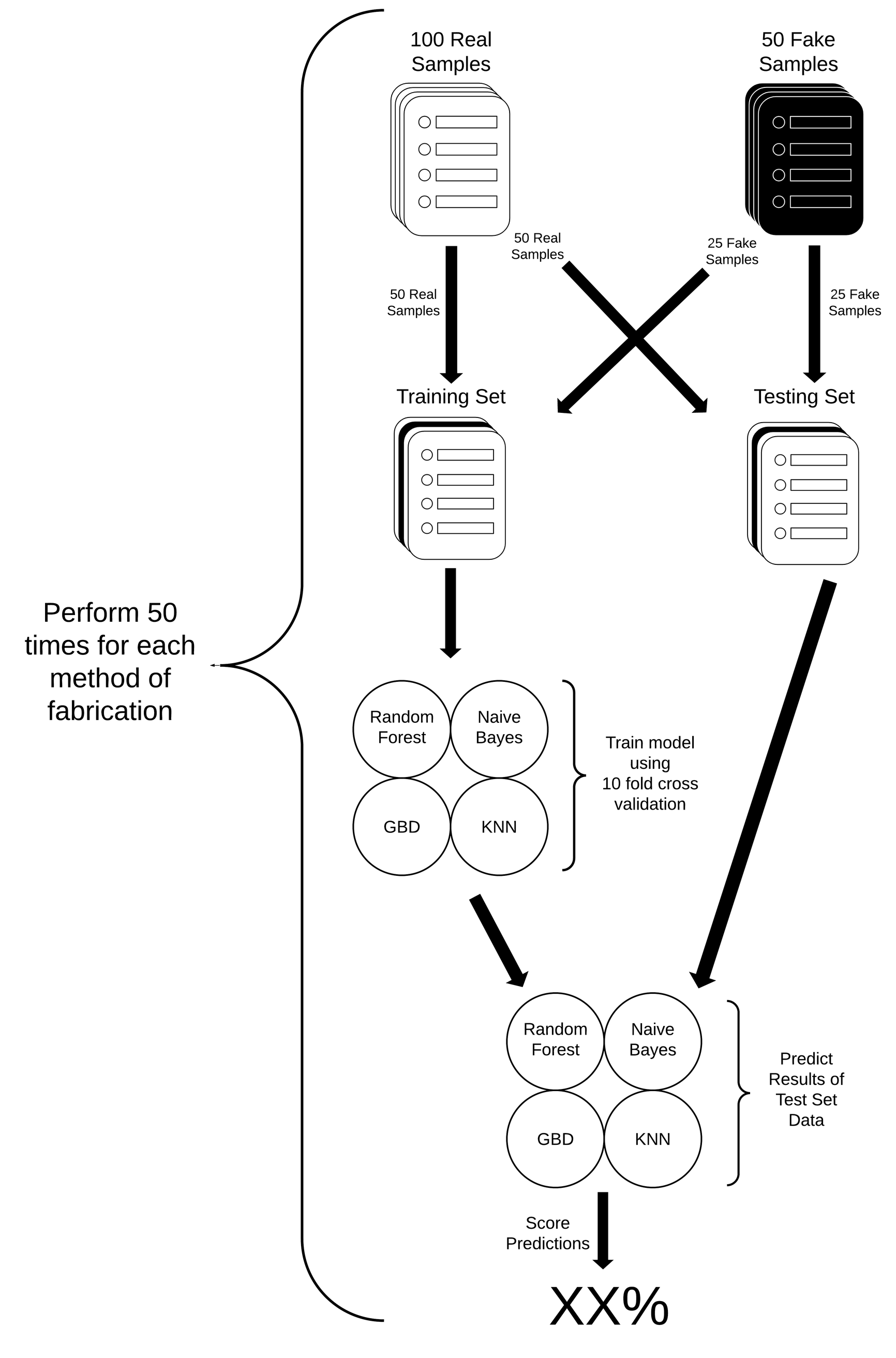

Supplement: S2 Fig — After creating 50 fake samples using any one of the three methods of fabrication, the 100 real samples and 50 fake samples were randomly split into a train and test set of equal size and proportions (50 real and 25 fake in each set). The training sets were then used to train various machine learning models using 10-fold cross validation. Next, trained models were used to make predictions on the testing data. Predictions were then scored with total accuracy. (TIF) [file pone.0260395.s002.tif]

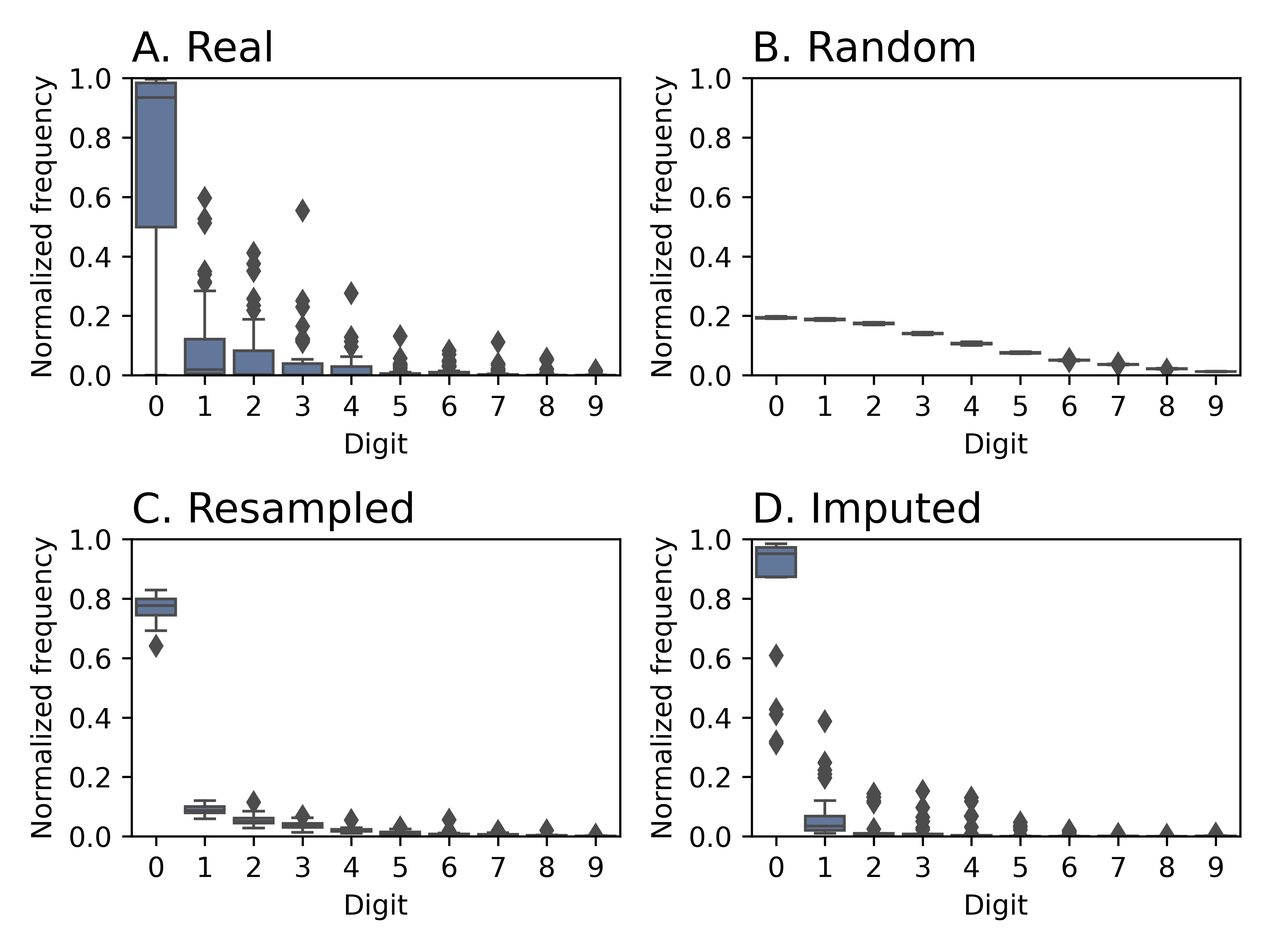

Supplement: S3 Fig — Distribution of normalized first-digit after the decimal frequencies in 75 real copy-number samples (A) and 50 fake samples generated by the random (B), resampled (C) and imputed (D) methods of fabrication. The x-axes represents each digit in the first position after the decimal place. The y-axes represents the normal frequency of the digit. Black lines represent the mean and diamonds represent outliers. Similar to what is seen in a distribution of first digits conforming to Benford’s Law, the CNA data also exhibits a long-right tail. (TIF) [file pone.0260395.s003.tif]

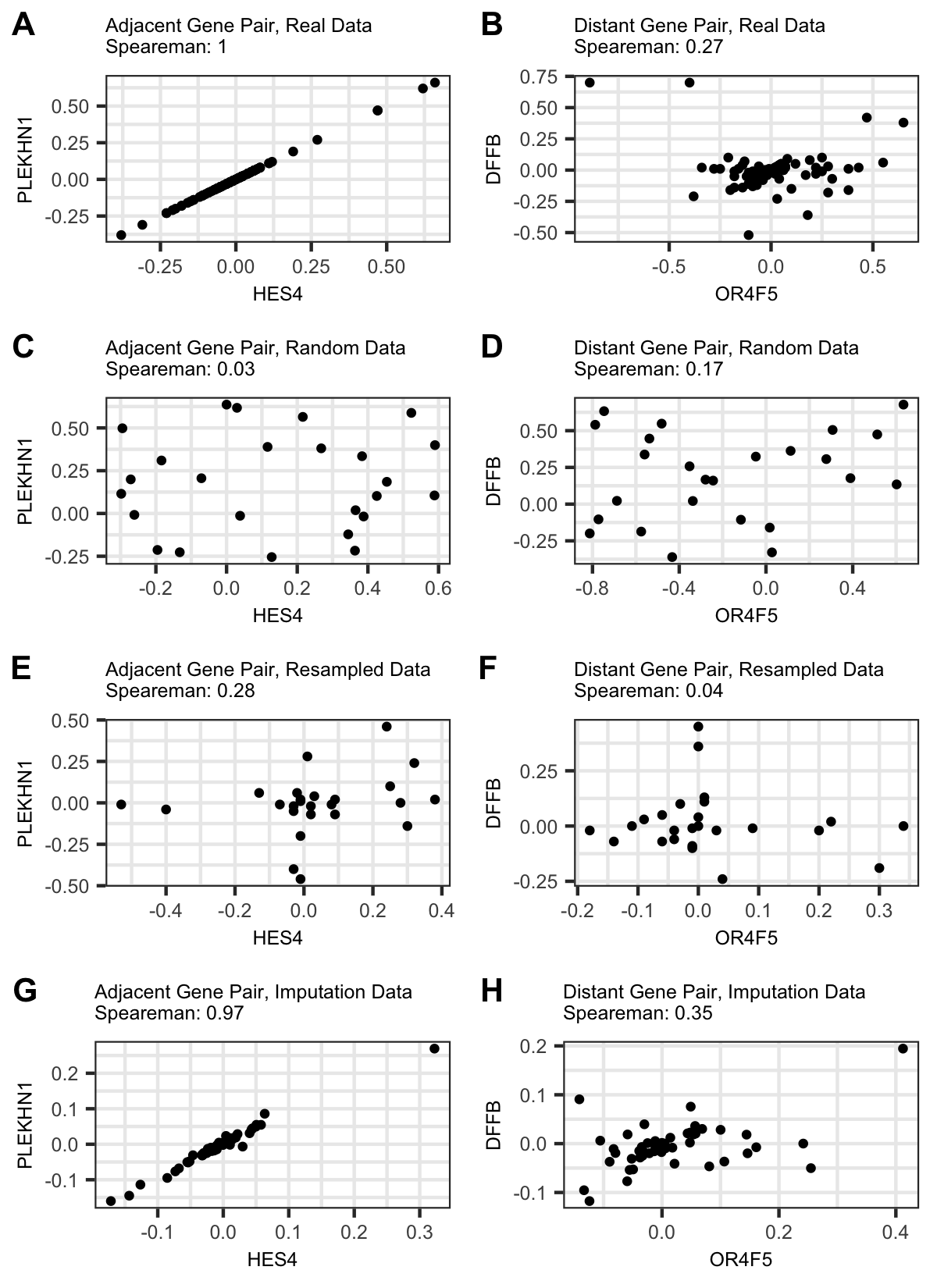

Supplement: S4 Fig — The correlation between pairs of genes is evaluated to determine whether fabrication methods can replicate inter-gene patterns. Plots on the left hand side (A,C,E, and G) display data from two correlated genes PLEKHN1 and HES4, adjacent genes found on 1p36. Plots on the right hand side (B,D,F, and H) display genes DFFB and OR4F5 gene with marginal Spearman correlation in the real data (.27). The plots reveal that random and resample data have little to no correlation between related genes. Imputation produces data with correlation values that are similar to the original data (.97 and.35, respectively). (TIF) [file pone.0260395.s004.tif]
