# Supplementary material for: Detecting fabrication in large-scale molecular omics data
Source: PLoS One. 2021 Nov 30;16(11):e0260395. doi: 10.1371/journal.pone.0260395 (PMC8631639; doi:10.1371/journal.pone.0260395)
Supplement: Attachment — Submitted filename: Response to Reviews.docx [file pone.0260395.s007.docx]

Response to Reviews

Below is a detailed response to reviewer critiques.

- Bradshaw and Payne

# Reviewer #1: 2nd review

----------

Reviewer #1 has asked for an extended discussion of two points. We have addressed each of these within the manuscript.

- Response 4: "the exploration of alternative methods is outside of the scope of our manuscript" --> I was expecting more a comment about that possibility than a deep exploration. One could discuss such issue based for example on the fact that k-NN (the less ML of the methods presented) also improves drastically (significantly :-) its predictive performance.

We have inserted some text that discusses this point.

- A comment I didn't think of on the first review that could enrich the discussion concerns the potential of using a similar approach, but in an unsupervised (or semi-supervised) manner for detecting "anomalies" in datasets so as to flag potential falsifications (as done in fraud or cyberattack contexts) without having a training set of already known fake-data strategies. (For future work, not to be addressed now)

We are grateful for this suggestion and have added a bit about the possibility of cluster and a couple citations to work on it in other fields.

Minor comments

We have fixed all the minor typographical errors noted

# Reviewer #3:

I love the idea behind this paper. Fraud is a very significant problem within scientific research, particularly for increasingly data right subject areas, and should be a concern for all of us in this community. Taking steps to develop tools to detect fraud is a key pillar of addressing this issue, alongside good Open Science practices that ensure transparency and replicability throughout the research chain (including in peer review!). Unfortunately, however, I have some significant concerns about this manuscript as it stand and I'm not convinced it's ready to be published as it stands. I outline those concerns below. First though, I would like to strongly encourage the authors to continue developing this manuscript, despite the significant review and publication delays since the first BioRxiv preprint. I have no doubt this will be a valuable piece of work in this important area.

Primary concerns:

1. The authors use three different mechanisms for generating fake data, random number generation, resampling and imputation. These are implemented with the aim of approximating the real data as accurately as possible. It is far from clear to me that any of these strategies reflect strategies scientists would actually use to fake data, however they are as reasonable as any other strategies given the lack of evidence base on this. The way they are used however is where my concern lies.

What motivation would someone have to fake data that reflects the real data and doesn't generate any clear 'result'?!? What the authors have here is a model that detects simulated data with the same characteristics as the current data. I suppose it is possible that scientists might want to fake (simulate) extra samples with similar statistical characteristics as their real data so as to inflate the sample number in their experiments, but it seems far more likely to me that scientists would try to fake data to generate a result.

For this paper, the simplest fake result to add to the data would be a shift in the CNV value to higher or lower values for specific subsets of samples or (more likely) for specific genes within specific subsets of sample. This more realistic test would be simple to implement within the three methods used here. To summarise; in order to be convinced that these methods are useful, I want to see their performance on a real world dataset with a CNV-treatment result in it, with different types of faked data (global up-/down- & specific gene up-down) added to either enhance/deplete the significance of the result, or to add new results to the data. I'd also like to see how models trained on the fake data with these signals in perform; retraining of the models here would probably require a more nuanced investigation for the training in order to avoid training the models just to recognise the up-/down-regulation, rather than the other characteristics of the fake data.

We appreciate the reviewer’s concern on this point and understand the fundamental question to be about whether our methods of making up data are realistic. We offer as a counter point two very high profile papers have been questioned. Both these papers make up data in a manner similar to our method. And the fraud is detected using digit frequencies. Therefore, we feel that our methods are realistic.

The first example, extensively explained here (https://datacolada.org/98), a PNAS paper is discovered to have fabricated half of their dataset. Moreover, and this is very important, the data can be observed to be fabricated based partially on a severe anomaly in the digit frequencies. The paper doubled the dataset by duplicating data points and introducing a strong signature discovered in digit frequencies.

The second and very recent example, extensively explained here (http://steamtraen.blogspot.com/2021/07/Some-problems-with-the-data-from-a-Covid-study.html), a COVID study was shown to be fabricated and contained duplicated patients. Again, one of the ways that fabrication was discovered and confirmed is through digit biases.

2. For the model trained on the two decimal digits, the models are essentially being trained to detect data that doesn't obey Benford's-law. The authors haven’t demonstrated that the machine learning models outperform the far simpler process of making the appropriate histogram and fitting a curve based on Benford's law to this and seeing if you get a decent fit (with a KS test, for example). It's possible that the ML models outperform this simple test, but the authors need to do this comparison to motivate the use of the more complex and opaque ML algorithms.

We do not claim to be identifying data that simply “doesn't obey Benford's-law”. We refer to our method as “Benford-like digit frequency”, it is inspired by Benford’s Law and takes the idea of digit frequencies but the digit distributions of real data in supplemental figure 3, while reminiscent of it, is not Benford’s distribution. Comparing our Benford-like digit-frequencies to the real distribution of Benford’s law is not a useful exercise. Additionally some of our ML models are no more complex than a KS test. We are not convinced that we need to prove one better than the other. We found a variety of methods that appear to work.

3. Figures 2 & 3 contain boxplots suggesting that some of the models have zero variation in their performance across different data subsets. In some cases this is because the clarifiers are apparently perfectly good/bad accuracy (which I am deeply suspicious of and seems too good to be true) but in some cases it's perfectly consistent accuracy performance (e.g. Fig 2 panel C). These results seem to be in disagreement with Figure 4 which suggests that the average accuracy performance never reaches 100% for any of the models. Something isn't right here. The authors need to carefully inspect their methods reconcile these figures, and either convincingly justify the perfectly good/bad/consistent performance or (more likely) fix the bug that’s causing these.

The largest x value used in Figure 4 was 17,000 (as opposed to the complete full number of features 17156) which can explain this change of accuracy. Because the analysis of Figure 4 depends on repeatedly randomly sampling a subset of features, using the true full set of features had to be excluded as there is only one way to pick 17156 features from 17156 features. Clarifying detail has been added that 17,000 was the max number of features used and an explanation as to why.

Detail comments:

1. Line 69/70. I think the readers would benefit from adding some clarity on the limitations of Benford's law here. In particular, it's only really valid for data that spans several orders of magnitude, and for data where the upper/lower limits are not tightly bounded.

Clarification added about the importance of spanning orders of magnitude and not being bounded

2. Line 86. I disagree with the statement that "making up data is always wrong"; a bit more nuance is needed here. Firstly, simulating data has a long history of being informative in many areas of science. Secondly, there is a grey area here around imputed data, and particularly the imputation of missing data. It is, for example, commonplace to model a covariate in order to impute missing values in this data, and then to use this covariate data - including the imputed data - in a second model which leads to interpretation. From a certain perspective (my perspective, for example!) this could be seen as 'making up data' that has a direct impact on results/conclusions (depending on the scale of the missing data). This is certainly not widely considered wrong or inappropriate.

We are grateful for pointing out this difficult description in the text. We are certainly not suggesting that imputation is wrong, and the reviewer is correct that it is widely used. Here we use algorithms which perform imputation for the purpose of inventing/fabricating datasets. As stated in the introduction, there is a nuance in the way we categorize this which depends on the author’s intent. We have added some new text to hopefully clarify our thinking.

3. Line 111-113: does this mean that some of the samples are represented twice in the 150 sample dataset, albeit with 10% imputed data? How do we know that the ML models are learning to separate the fake samples from the real based on the imputed data signal, rather than needing both a duplicated sample and the imputed data signal. If you added samples that aren't in the original data, with a 10% imputation, would the performance of the models be as good?

We are again grateful to the reviewer for pointing out this section and how it needs to be clarified. In our method, the samples that are fabricated via imputation are composed of 100% fabricated imputation data. The data was just nullified and imputed in chunks of 10%, repeated 10 times so all the data in the final fake sample was fake and unique to it. Doing it in chunks like this was a necessary adjustment to meet the expectations of missForest (plus it sped up an extremely slow process). Additional detail has been added to this section.

4. Line 166: "Machine learning cannot…" I know what you're getting at, but this is not well worded. I think you want to say something like : "Trained ML models are restricted to data that conform to the model input specifications (i.e. the same number of input features, for example).

Thank you. We have adopted your explanation.

5. Line 168: I think it would be worth noting here that the generalizability of this model comes with a cost - it will only work for data where Benfords-law should be valid, which is certainly not all datasets.

I have added a clarifying statement about this “though it’s effectiveness will still be dependent on the existence of digit-frequency patterns”

6. Line 177: "tiddy-verse" should be "tidyverse"

Fixed

7. Line 233: "six" this should be five - this needs checking throughout the paper since the number of models used has changed through the review process.

Fixed, the other review caught this too.

8. Line 247: "The remaining four models…". I think this region of text has been re-ordered quite a bit during the review process and it doesn't make much sense now since we haven't has the results for the fifth model yet at this point. I think the authors need to give this section a careful read and make sure it flows sensibly.

Yes, some mistakes were made and not caught after our first round of revisions (number mismatch and confusing use of “remaining”). Changes have been made to this paragraph and are highlighted.

9. Line 286: "[29149684]" I think this should be a reference??

Yep, thanks

10. Line 289: "While Benford's law…" The shift to use the decimal point digits rather than the leading digit is necessary because of the constraint that Benford law works (best) for numbers spanning several orders of magnitude. This is not the case for the first digit in the CNV data, but this value is usually a non-zero value so the first and second digits necessarily span orders of magnitude. This is a dataset specific approach though. in a dataset comprised mainly of numbers between 0 and 0.09, you would need to use the third and fourth decimal point digits. This would be work illuminating here.

These details have been added.

11. Line 301-307: "Machine learning typically…" repetition of previous text and discussion. I think this can be removed.

Agreed and removed. Brevity is better

12. Line 339. I'm not very surprised at the reasonable performance with as few as 10 genes here. 10 genes x 75 samples = 750 datapoints. This is plenty to build a histogram to compare with the Benfords Law curve (the equivalent of which is what the ML models are learning to do) .

13. Line 353: I think "per data point" should be "per sample" here.

Changed

14. Figure 2: I can't really see the details of this figure well - the resolution is quite low in the PDF embedding. It would be useful to explain the components of the box plot (median, quartiles, indents, etc) for those not familiar with boxplots.

Sorry the resolution was low, our figures were all saved and submitted with fairly high resolution. I assume some resolution was lost in the PDF embedding process but hope that is not the case in the actual publication. If the next figures still look fuzzy you can find all the actually .png and .tiff files properly labeled and in the Figures directory on the Github repo: <https://github.com/MSBradshaw/FakeData/tree/master/Figures>

15. Supp. Fig. 3. This figure is really useful (I'd put it in the main paper) but it's a nightmare to read because it's very busy. I suggest that the authors split the figure into four facet panels with one dataset per panel.

Supplemental Figure 3 has been split in two a 4 panel plot
